# Do Super-hydrophilic Surfaces Affect Implant Primary Stability in the Early Healing Phase of Osseointegration? A Systematic Review with Metanalysis

**DOI:** 10.3290/j.ohpd.c_2235

**Published:** 2025-08-25

**Authors:** Luigi Canullo, Maria Menini, Luca Guardone, Valeria Merlini, Virginia Cameroni, Anton Sculean, Paolo Pesce, Massimo Del Fabbro

**Affiliations:** a Luigi Canullo Associate Professor, Department of Surgical Sciences (DISC), University of Genoa, Genova, Italy; Department of Periodontology, University of Bern, Bern, Switzerland. Conception, design, investigation, data collection, drafting and critical revision of the article, read and agreed to the published version of the manuscript.; b Maria Menini Associate Professor, Department of Surgical Sciences (DISC), University of Genoa, Genova, Italy. Critical revision of the article, read and agreed to the published version of the manuscript.; c Luca Guardone Dental Student, Department of Surgical Sciences (DISC), University of Genoa, Genova, Italy. Data collection, data curation, methodology and drafting of the article, read and agreed to the published version of the manuscript.; d Valeria Merlini Dental Student, Department of Surgical Sciences (DISC), University of Genoa, Genova, Italy. Data collection, data curation, methodology and drafting of the article, read and agreed to the published version of the manuscript.; e Virginia Cameroni Dental Student, Department of Surgical Sciences (DISC), University of Genoa, Genova, Italy; Department of Periodontology, University of Bern, Bern, Switzerland. Data collection, data curation, methodology and drafting of the article, read and agreed to the published version of the manuscript.; f Anton Sculean Professor, Department of Periodontology, University of Bern, Bern, Switzerland. Reviewed and edited the manuscript, read and agreed to the published version of the manuscript.; g Paolo Pesce Associate Professor, Department of Surgical Sciences (DISC), University of Genoa, Genova, Italy. Reviewed and edited the manuscript, read and agreed to the published version of the manuscript.; h Massimo Del Fabbro Full Professor, Department of Biomedical, Surgical and Dental Sciences, Università degli Studi di Milano, Milan, Italy; Fondazione IRCCS Ca’ Granda Ospedale Maggiore Policlinico, Milan, Italy. Supervision and statistics, read and agreed to the published version of the manuscript.

**Keywords:** bioactive surface, dental implants, implant primary stability, implant survival, marginal bone loss, osseointegration

## Abstract

**Purpose:**

Implant stability, related to mechanical (primary) and biological (secondary) bone-to-implant interactions, is essential for osseointegration. Implant surface bioactivation is a process designed to accelerate and enhance surface-cell interaction.The purpose of this systematic review was to determine whether a beneficial effect of bioactive (BS) over traditional surfaces (TS) can be identified.

**Materials and Methods:**

An electronic search of Pubmed, Scopus, and CENTRAL databases was performed to identify randomized (RCT) and non-randomized controlled trials comparing BS and TS implants. Risk of bias was assessed using the Cochrane Collaboration tool for RCTs and the Joanna Briggs Institute tool for non-RCTs. Outcome variables were implant stability quotient (ISQ) measured through resonance frequency analysis from placement to prosthetic loading, one-year implant survival rate, and marginal bone loss (MBL). Meta-analysis was performed where possible.

**Results:**

Of the 6920 records identified, 13 RCTs and two non-RCTs were included, reporting on 1256 implants (49.8% TS and 50.2% BS) in 596 patients. Four of the studies had a low risk of bias, three had a moderate risk and eight had a high risk. The meta-analysis showed no evidence of an effect of implant surface on survival rate (p = 0.99, 10 studies) and MBL (p = 0.86, 5 studies). At baseline (10 studies) and at one month (9 studies) the ISQ did not differ statistically significantly different between groups. A statistically significantly greater increase in ISQ was found for the BS implants compared to the TS implants (p = 0.04) at three months after placement (9 studies).

**Conclusion:**

An advantage of BS over TS during the early osseointegration phase could not be demonstrated, but a positive effect on implant stability seems to occur after three months of placement. The statement that bioactive surfaces may safely allow early and immediate implant loading is insufficiently supported by the current evidence.

Implant primary stability is determined by the friction between bone and implant at the time of insertion and tends to decrease as existing bone is progressively replaced by new bone at the implant-bone interface during osseointegration.^23^ Secondary stability, on the other hand, is determined by the osseointegration process: adhesion of the osteoblastic cells occurs at the level of the implant surface, causing progressive deposition of bone tissue and consequent implant stabilization.^32,45,36^ At the beginning of the osseointegration, only early osteoblast adhesion to the implant surface occurs. However, this alone is not sufficient to ensure complete healing. For this to happen correctly, precursor osteogenic cells need to proliferate and differentiate and then adhere appropriately to the implant surface. To facilitate this process, some features of the implant surface such as roughness, wettability and hydrophilicity are fundamental.^30,63^ These surface changes can be achieved by subtractive techniques such as sandblasting and acid-etching.^20,67,39^

Today, the process of osseointegration can also be facilitated by the use of implants with innovative bioactive surfaces characterized by hydrophilicity, which may favor proper osseointegration.^18,73^

There are various procedures to make implant surfaces bioactive, one of which is photofunctionalization.^13,15^ This technique is used to prevent the aging of titanium, which would otherwise be an obstacle to effective osseointegration. Photofunctionalization transforms the hydrophobic surface into a super-hydrophilic surface and allows the surface of the implant to be cleansed of the hydrocarbons formed by the aging of titanium.^54^ To achieve this, the implants are functionalized by treating them with a UV light for 15 minutes using a chairside photo-device. These 15 minutes of UV treatment are followed by a further 5 minutes of clean-ozone treatment. All this is done immediately before the implant is placed.

More recently, SLActive implants have been introduced.^15,40^ The surface of these implants is obtained by chemical modifications, such as creating a hydroxylated/hydrated TiO_2_ film, which in turn creates high surface energy, as performed using N_2_ carrier gas. This high surface energy is maintained by storage in isotonic saline.

Hydrophilicity, or “wettability,” is a fundamental characteristic in the processes that affect the osseointegration capacity of an implant, whether it is used for orthopedic or dental purposes.^7,59,60,65^

Hydrophilicity is a characteristic that affects the implant surface, and is a direct consequence of titanium aging, influencing biological processes such as the speed and quality of integration between bone and implant.

Most implant processing techniques produce surfaces that are initially super hydrophilic, with an initial angle between water and the implant surface between 0 and 10 degrees. However, this angle tends to increase over time. A surface is considered hydrophilic when the angle between the surface and an overlying drop of water is between 10 and 30 degrees, hydrophobic when the angle is between 30 and 90 degrees, and water repellent (hydrophobic) when the angle is greater than 90 degrees.^8,21^

In-vitro studies have repeatedly shown that this property can greatly influence the adhesion between the cells involved in the process of osseointegration and the implant surface.^29,68^ However, the results of in-vivo human studies are conflicting and do not clearly indicate a net advantage in the use of bioactive implants, which, despite their high hydrophilia, appear to osseointegrate as well as standard implants.

Depending on how the implant surface is treated, it will age differently. This phenomenon has been investigated in a recent study, which showed, for example, that an acid-etched surface already becomes hydrophobic seven days after production, with a water-titanium angle of 50 degrees.^58^ Conversely, the sandblasted surface is still highly hydrophilic after seven days. This is due to the speed at which a film of hydrocarbons is deposited on the titanium surface, reducing the wettability. This film of hydrocarbons is formed because the container used for storage, although sterile, contains oxygen and other contaminants that, when in contact with the implant surface, make it hydrophobic.^42^ This study has also shown that the aging of titanium is more or less reversible depending on the treatment to which it is subjected, the most suitable being UV, which immediately makes the implant surfaces super hydrophilic, restoring an angle of 0 degree between the implant and water and eliminating the hydrocarbon film.^42^

However, recent reviews of the literature have failed to demonstrate a clinical advantage of these bioactivated surfaces.^18,73^

Resonance Frequency Analysis (RFA), a widely used technique for measuring the stability of the bone-implant complex, may provide an indirect estimate of implant osseointegration.^12,57^ RFA quantifies the frequency of oscillation of the implant within the bone in response to an pulse of known frequency. The unit of measurement is the Implant Stability Quotient (ISQ). The ISQ values range between 0 and 100; ISQ values greater than 70 are considered excellent, indicating implant osseointegration and the ability to sustain functional loading.^52^

The aim of this study was to conduct an updated systematic review of the literature on the performance of implants with bioactive surfaces (BS) compared to traditional surface (TS) implants, focusing on clinical and radiological outcomes.

## MATERIALS AND METHODS

### Protocol and Registration

This review followed the guidelines of the Preferred Reporting Items for Systematic Reviews and Meta-Analyses (PRISMA). The protocol was registered on PROSPERO (N. CRD42023433722).

### Data Sources 

Detailed search strategies were conducted in the electronic databases PubMed, Scopus and Cochrane CENTRAL, with language restrictions (articles written in English only) and included articles published up to June 2024. Specific keywords, combined using the Boolean operators AND, OR were used to search the database for studies of interest. The search string for PubMed was (“surface modification” OR “bioactive surface” OR “active surface “ OR “hydrophilic surface” OR “plasma” OR “ ultraviolet” OR “wettability” OR “Functionalization” OR “ultra-hydrophilic” OR “Photocatalysis” OR “Ultraviolet photofunctionalization” OR “Glow discharge” OR “UV”) AND (“Dental implant*”).

### Eligibility Criteria 

The following criteria were considered for inclusion:

Population: patients who received implant treatment;
Intervention: patients who received UV-treated implants or implants with the surface modified to increase the wettability and improve osseointegration (e.g., sandblasted surface, acid-etched (SLActive) implant) (BS, bioactive surface);
Comparison: patients who received implants with untreated (traditional) surface (TS);
Outcome: implant survival rate, marginal bone loss, implant stability;
Study design: randomized controlled trials (RCTs) and controlled prospective studies.
Time: all studies with at least 3-month follow-up

The authors excluded in-vitro studies, in-vivo studies on animal models, retrospective studies, studies without a control or test group, and studies focused on coated implant surfaces. There were no limitations regarding the year of publication.

### Study Selection

Two reviewers (L.G., V.C.) independently selected the relevant studies. The first screening was based on the title and abstract of the studies retrieved from the electronic search. All studies non-pertinent were excluded. The full text of all the eligible studies at this stage was obtained and evaluated to ensure the paper met the inclusion criteria. For all the studies excluded at this stage, the reason for exclusion was noted. Any discordance among the reviewers was resolved by discussing with a third reviewer (L.C.). Agreement between reviewers was assessed using the Cohen κ coefficient, where κ was at least 0.85.

### Data Extraction

Data extraction was performed independently by two reviewers (LG, VM) and included from each study the authors, study design, year of publication, dental implants surface treatments and modification methods, implant stability quotient (ISQ), implant survival rate, marginal bone loss (MBL), implant location in the mouth, and probing depth (PD). The data were divided into two groups according to the implant surface: bioactive (BS) and traditional surface (TS).

### Risk of Bias Assessment

Two reviewers (VM, VC) independently performed the risk of bias assessment of the included studies. The latter was based on seven parameters: random sequence generation (selection bias), allocation concealment (selection bias), blinding of participants and personnel (performance bias), blinding of outcome assessment (detection bias), incomplete outcome data (attrition bias), selective reporting (reporting bias) and other potential sources of bias (sample size calculation, estimation of homogeneity between treatment groups at baseline). Based on the traditional Cochrane Collaboration risk-of-bias tool for RCTs, a study was considered at low risk of bias if all seven parameters were at low risk of bias, moderate risk of bias if there was unclear risk of bias of at least 1 parameter, and high risk of bias if at least 1 parameter was scored as being at a high risk. For the non-RCT studies, the Joanna Briggs Institute checklist for cohort studies was used. Studies were judged at critical risk of bias if one or more items were critical, at serious risk if more than 4 items were unclear, at moderate risk if 2 to 4 items were unclear, and at low risk if there was no more than one item unclear. In case of doubt or discrepancies, a third reviewer was consulted (MDF).

### Outcome Measures

The objective of the present research was to compare the results of ISQ, survival rate and MBL between TS implants (control) and BS implants (test). ISQ, or Implant Stability Quotient, measured through the analysis of the resonance frequency, is a physical parameter indicating the primary stability of a dental implant. Resonance frequency analysis is a quantitative, diagnostic and noninvasive method used for determining implant stability. ISQ ranges from 1 to 100 and is an estimate of the stability of the implant. Higher ISQ values indicate better implant stability and osseointegration. For this review, since the stability of implants might be assessed at different times after placement, it was decided to consider ISQ measured at baseline, then after one month (with a tolerance of 1 week), and finally three months after placement (with a tolerance of 2 weeks).

Implant survival rate was estimated at the implant level. It is represented by the proportion of implants still in function at a follow-up of at least 12 months with respect to implants inserted at baseline, lacking biological complications at the hard and soft tissue levels.

MBL is defined as a loss in the apical direction of alveolar bone surrounding the dental implant in relation to the marginal bone level initially detected at the time of implant placement. At 1 year following placement, an implant should have <0.2 mm annual loss of marginal bone level to satisfy the criteria of success.^35^

### Statistical Analysis

To estimate the overall effect for the outcomes investigated when at least two studies with similar outcomes and protocols were found, pairwise meta-analysis was undertaken using Review Manager (RevMan Version 5.4.1, The Cochrane Collaboration, 2020). A fixed-effects model was first applied, and if significant heterogeneity among studies was detected, a random-effects model was implemented. Heterogeneity among the included studies was assessed using Cochran’s test for heterogeneity, and the significance threshold was set at p < 0.1. In case of marked heterogeneity, sensitivity analysis was performed by excluding studies with a high risk of bias or non-RCTs. For quantitative variables, the intervention’s effect was calculated as the mean difference (MD) together with 95% confidence intervals (CIs). If a single study’s weight was greater than 80%, standardized mean difference (SMD) was used. To address possible missing standard deviations, the methods outlined in Section 7.7.3 of the Cochrane Handbook for Systematic Reviews of Interventions, Version 5.1.0^46^ were used when applicable. For variables expressed as proportions, the effect was estimated as the risk ratio (RR) together with 95% CIs. A p-value < 0.05 was considered statistically significant.

## RESULTS

The data are available upon request from the corresponding author.

**Fig 1:**
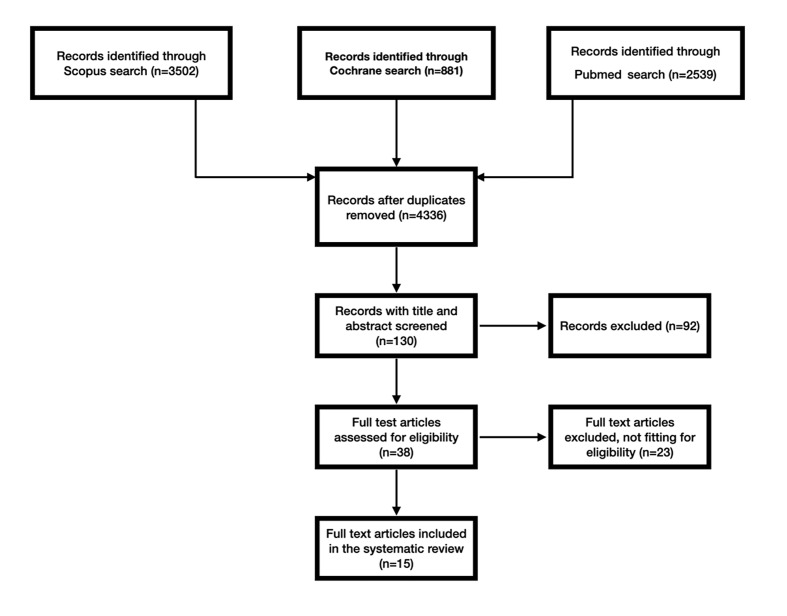
Flowchart of the selection process.

The electronic search produced a total of 6920 articles. Figure 1 illustrates the selection process. After initial screening based on title and abstract, 38 studies were considered eligible, and the full text was obtained and evaluated to assess whether the studies met the inclusion criteria.

All studies excluded at this stage are listed in Table 1, together with the reason for exclusion.^3-6,16,17,19,22,24,26,27,37,38,41,43,48,49,50, 63,68,72,74^ Finally, 15 studies (13 RCTs and 2 non-RCTs), reporting on 1256 implants (49.8% TS and 50.2% BS) in 596 patients were included.^1,2,10,14,28,33,34,66,44,47,53,55,56,61,62^ The main characteristics of the included studies are summarized in Table 2.

**Table 1:** List of excluded studies and reason for exclusion

Study	Reason for exclusion
Stricker et al, 2003	No control group
Bornstein et al, 2005	No control group
Ganeles et al, 2008	No control group
Zollner et al, 2008	No control group
Bornstein et al, 2009	No control group
Morton et al, 2009	Not a prospective comparative study
Bornstein et al, 2010	No control group
Loungo et al, 2010	Not a prospective comparative study
Bosshardt et al, 2011	No control group
Lang et al, 2011	The outcomes considered do not match with our focus
Nicolau et al, 2011	No control group
Filippi et al, 2013	Not a prospective comparative study
Funato et al, 2013	Not a prospective comparative study
Guler et al, 2013	Does not provide separate ISQ data for BS and TS implants
Markovic et al, 2014	No control group
Hicklin et al, 2015	Not a prospective comparative study
Wallkamm et al,2015	No control group
Kitajima et al, 2016	No control group
Yamaner et al,2017	Not a prospective comparative study
Hirota et al, 2018	Not a prospective comparative study
Nicolau et al, 2019	No control group
Hicklin et al,2020	No control group
Hirota et al, 2020	Multiple publication


**Table 2:** Characteristics of included studies

Included study	Study design	Split mouth	Test (BS)	Control (TS)	Patients BS	Implants BS	Patients TS	Implants TS	Follow-up, months	Implant location (no. of implants)
Novellino et al, 2017	RCT	Yes	Modified SAE	SAE	21	32	21	32	12	Posterior maxilla (64)
Choi et al, 2021	RCT	No	UV-treated	SAE	18	29	16	28	12	Posterior maxilla (57)
Hirota et al, 2016	CCT	Yes	UV-treated	Untreated	4	25	5	24	24	Various sites (49)
Karabuda et al, 2010	RCT	Yes	Modified SLA	SLA	22	48	22	48	12	Maxilla (57) Mandible (39)
Shah et al, 2021	RCT	NO	PF pre-treated	Untreated	27	27	28	28	12	Anterior maxilla (55)
Markovic et al, 2016	RCT	yes	SLActive	SLA	20	40	20	40	12	Anterior maxilla (25), posterior maxilla (17), anterior mandible (10), posterior mandible (28)
Puisys et al, 2019	RCT	Yes	UV-treated	untreated	180	180	180	180	24	Maxilla (142), mandible (218)
Nack et al, 2015	RCT	Yes	SLActive	SLA	20	49	20	48	60	Various sites (97)
Filho et al, 2018	RCT	Yes	SLActive	SLA	19	19	19	19	3	Posterior mandible (38)
Ozel et al, 2021		Yes	SLActive	SLA	12	25	12	25	3	Various sites (50)
Barbosa et al, 2021	RCT	Yes	DASH	DAS	20	20	20	20	3	Posterior maxilla (40)
Park et al, 2010	RCT	No	RBM treatment	SLA	28	39	28	36	12	Posterior mandible (75)
Sandhu et al, 2021	RCT	Yes	UV-treated	untreated	34	34	34	34	12	Posterior jaws (68)
Canullo et al, 2024	CCT	No	MultiNeO NH CS	MultiNeO CS	18	30	18	30	6	D3 and D4 (poor density bone) (60)
Rani et al, 2024	RCT	No	Uv-treated	Standard implants	30	33	30	34	12	Various sites (67)
BS: bioactive surface; TS: traditional surface; RCT: randomized controlled trial; CCT: controlled clinical trial; SAE: sandblasted and acid-etched; DAS: double acid-etching and sandblasting; DASH: DAS plus stored in 0.9% saline solution to increase hydrophilicity; RBM: hydroxyapatite–Ca_10_-(PO_4_)6(OH)2 spraying on the implant surface to set the surface roughness (Ra) to 1.2–1.8 mm; PF: photofunctionalization.

### Risk of Bias

**Fig 2:**
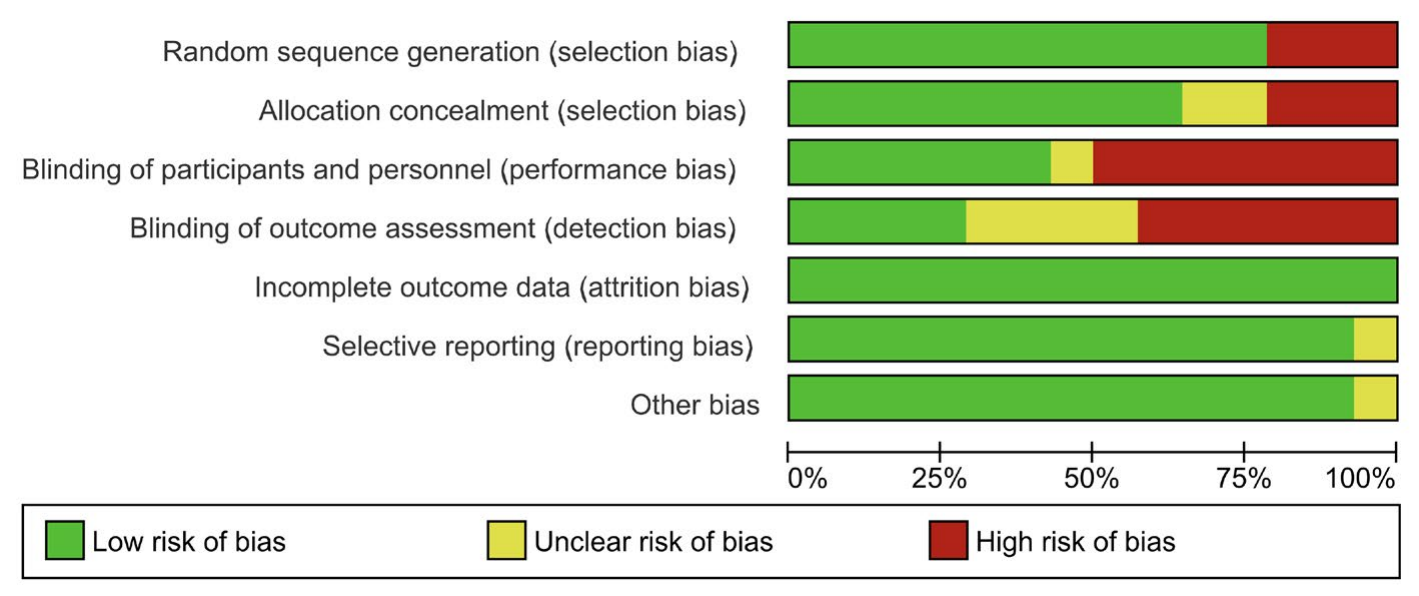
Risk of bias graph, showing the overall risk for each item considered. Blinding was the item with the greatest risk of bias.

Figure 2 summarizes the overall risk of bias for each item, and Figs 3a and 3b represent the risk of bias of the individual randomized studies. Four randomized studies were judged at low risk of bias, three at moderate risk, and 7 at high risk of bias. The only non-randomized study was judged at serious risk of bias.^28^

On the other hand, the only non-randomized study^28^ was judged at critical risk.

### Implant Stability Quotient

**Fig 4:**
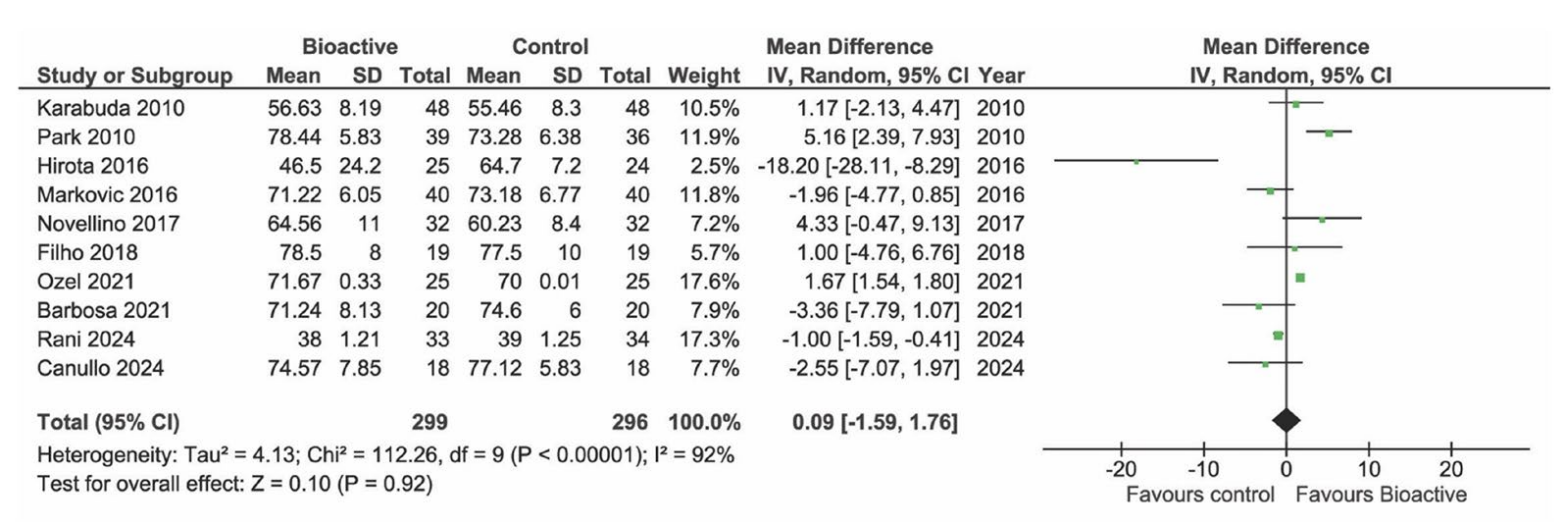
Forest plot of studies reporting ISQ at baseline.

**Fig 5:**
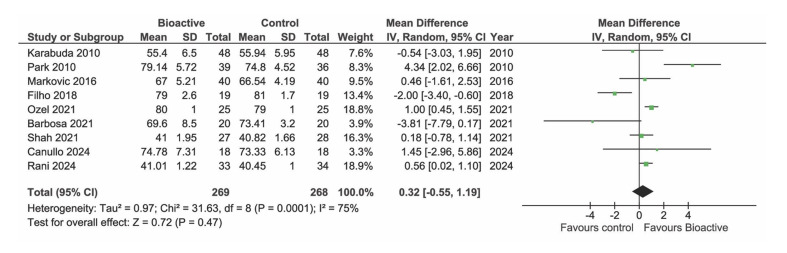
Forest plot of studies reporting ISQ at 3–5 weeks.

**Fig 6:**
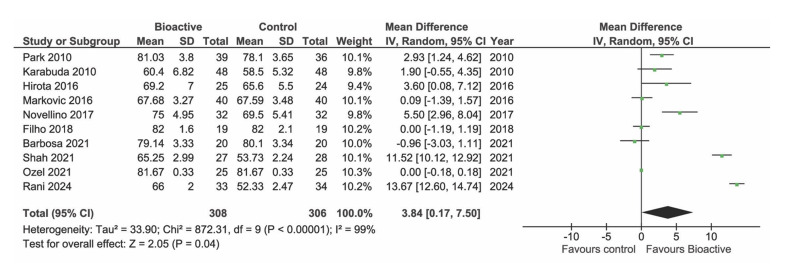
Forest plot of studies reporting ISQ at 10–14 weeks.

Of the included studies, nine reported data on ISQ. However, one study^65^ could not be included in the meta-analysis because it only provided follow-up ISQ values and not the baseline value. At baseline (Fig 4, 8 studies) and at 1 month (Fig 5, 7 studies) there were no statistically significant differences in ISQ between BS and TS group (p = 0.19 and p = 0.79, respectively). One study^28^ appeared as an outlier at baseline due to very low ISQ values. These studies included both regular cases and complex cases (implant placed in grafted sinuses), with the latter contributing to a decrease in the mean ISQ value. However, even excluding this study, the difference between the BS and TS group remained non-statistically significant at baseline (data not shown). At three months after placement, a statistically significantly greater increase in ISQ was found in the BS group compared to TS implants (p = 0.03) (Fig 6, 8 studies).

### Implant Survival

**Fig 7:**
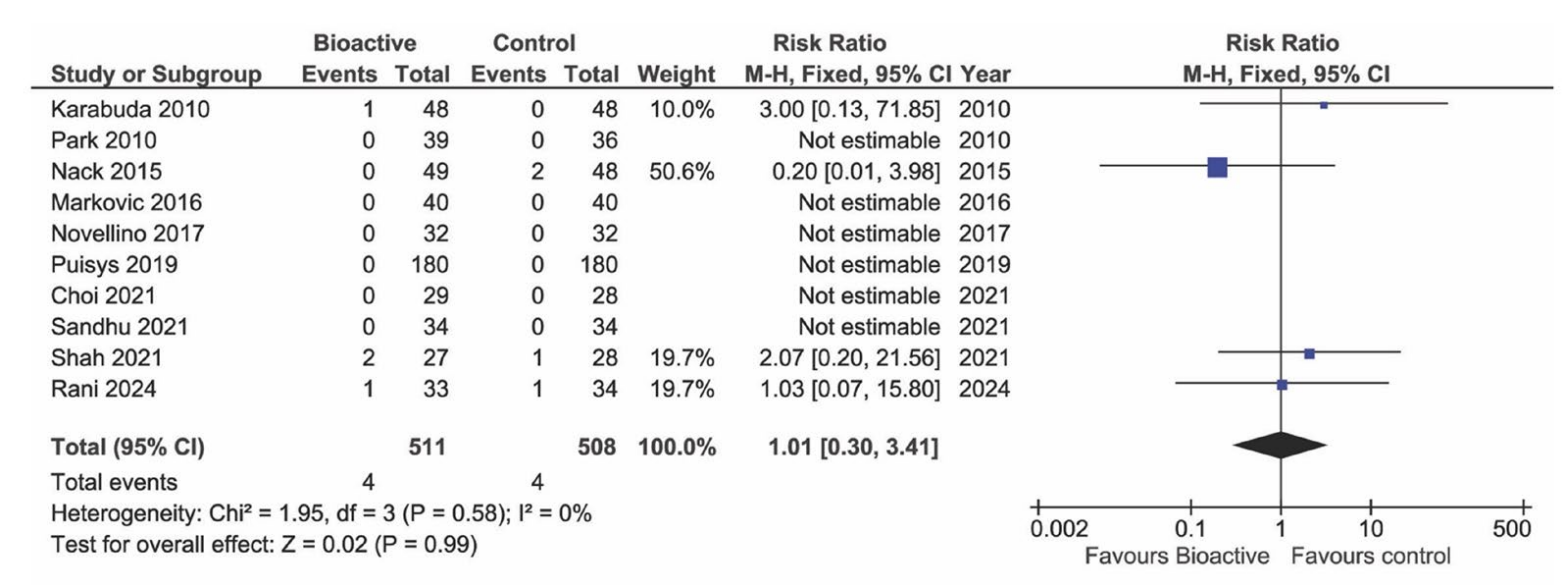
Forest plot of studies reporting implant survival after at least 1 year.

The meta-analysis of studies with a follow-up of at least one year showed no evidence of an effect of implant surface on the survival rate (Fig 7, p = 0.99, 10 studies, 498 BS implants and 494 TS implants). In seven studies, no failure occurred in either group.

### Marginal Bone Loss

**Fig 8:**
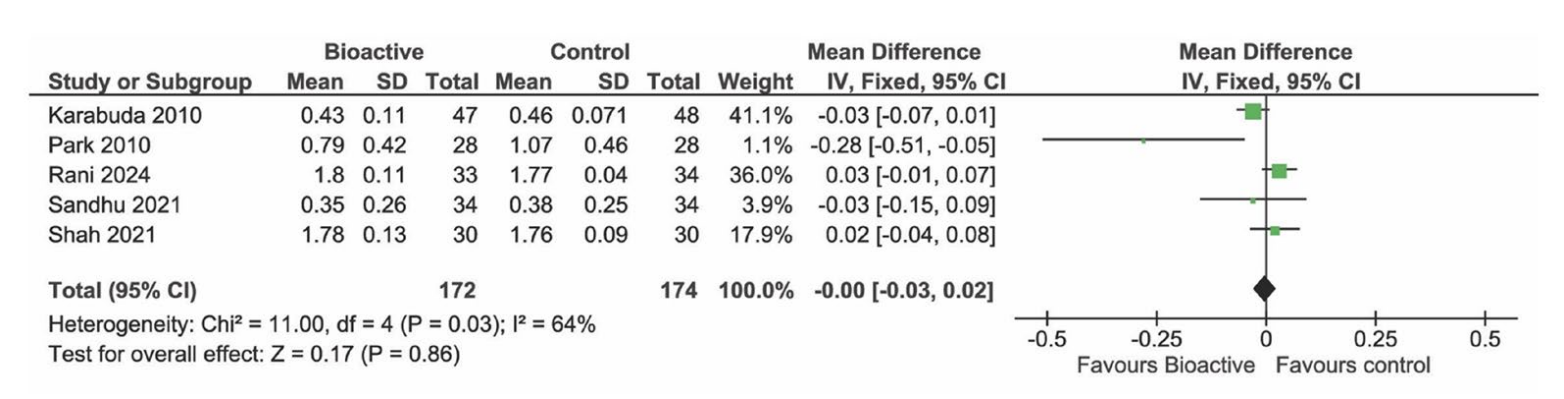
Forest plot of studies reporting marginal bone level change after at least 1 year.

Five studies were included in the meta-analysis of MBL (Fig 8). There was no evidence of an effect of the surface type (p = 0.11, MD= -0.02 mm, 95% CI -0.05, 0.01, 330 BS implants and 328 TS implants).

## DISCUSSION

In the present systematic review, the difference in the osseointegration process between standard implants (not subjected to bioactivation) and bioactive implants (SLActive or UV Ray-treated implants) was investigated.

Analyzing the available data by parameters such as MBL and survival rate, no substantial differences were found in the long-term follow-up of osseointegration between control and test group. However, analyzing the ISQ data, a trend in favor of bioactive surfaces in the early healing phase (around 1 month) was apparent that became statistically significant at the 3rd month.

On the one hand, the results regarding clinical outcomes appear to be comparable to those reported in previous systematic reviews.^70^ However, on the other hand, a statistically significant difference between bioactivated and traditional surfaces in terms of stability outcomes was found.^2,44,55,62,70^

Osseointegration occurs through cellular stratification observed at the implant surface, typically reaching its peak around the 8th week. This biological process defines secondary stability, also referred to as biological stability. This progression depends on the prior establishment of primary stability, also known as mechanical stability, which is largely influenced by the thickness of the cortical bone.^34,41^

Ideally, bioactive implants are intended to accelerate secondary stability, and therefore osseointegration, by promoting the interaction between bone marrow cells and the implant surface.^2,53,61^

The ISQ is a parameter describing the stability of the implant inside the bone by measuring via resonance frequency analysis. The results of the ISQ extrapolated from the literature analyzed in this review showed a positive trend in favor of bioactive implants beginning at one month from insertion, although this was not statistically significant.

One possible explanation for these findings could be that part of the implant sites analyzed in the selected articles were located in the posterior mandible. As matter of fact, the loading phase’s stability is strongly influenced by the bone density of the implant site itself. Bone types D1 and D2 have a thick cortical conformation, resulting in a saturation effect of bioactivation on the implant surface. It was known that in a prevailing cortical area (bone density D1/D2), especially in the early healing phase, stability is mainly primary. This clinical scenario might jeopardize the efficacy of the bioactive implants due to the paucity of medullary tissue that instead promotes secondary stability. On the other hand, presence of a wider volume of medullar bone in contact with the implant surface (D3/D4) could exploit the bioactivation effect, favoring a greater interaction with the cells of the bone marrow and thus determining a clear improvement of secondary or biological stability.

Due to the presence of confounding factors (stability mainly due to the cortical component) in this early healing phase, a significant advantage in the use of bioactive surfaces is not detectable, even if a favorable trend is apparent. As opposed to the previously published reviews, outcomes reported in the present study presented a trend at the early stage which became statistically significant in the longer follow-up due the higher number of studies included.

In fact, one systematic review/meta-analysis^1^ included fewer articles and stopped the observation period at week eight. Eventually, the lower amount of data combined with a shorter observation period might explain the difference between results reported by Almassri et al^1^ and the present study. The advantage of bioactive implants found in the present review as of the tenth week may be related to their ability to anticipate and promote secondary stability.

Analyzing the data related to marginal bone loss, the results showed a slight advantage in favor of bioactive implants, although this difference did not reach statistical significance. This may be due to the inability of bioactivation to withstand the effects of prosthetic loading.^1^ As for the MBL, the difference in survival rate between the two groups considered is minimal and slightly favors the bioactive implants,^1,44,47,62^ confirming the results of a previously published systematic review.^13^ In that study, the comparison of the two surfaces based on survival rate showed a positive trend towards bioactive implants.^13^ This might also help prevent peri-implant disease.^71^ Additionally, other clinical aspects, such as the morphology of the abutments, must be considered.^9,11^

## CONCLUSION

An advantage of BS over TS during the early osseointegration phase could not be demonstrated, but a significant positive effect on implant stability seems to occur after three months of placement. The statement that bioactive surfaces may safely allow early and immediate implant loading is insufficiently supported by the current evidence and may need further studies. In fact, to substantiate the potential benefits of bioactivation, studies should specifically target implant sites with a higher prevalence of medullary bone (D3-D4). This would enable a comparison between bioactive and non-bioactive surfaces, where the effect of bioactivation on stability is not diminished by the predominance of cortical bone.

## REFERENCES 

**Fig 3:**
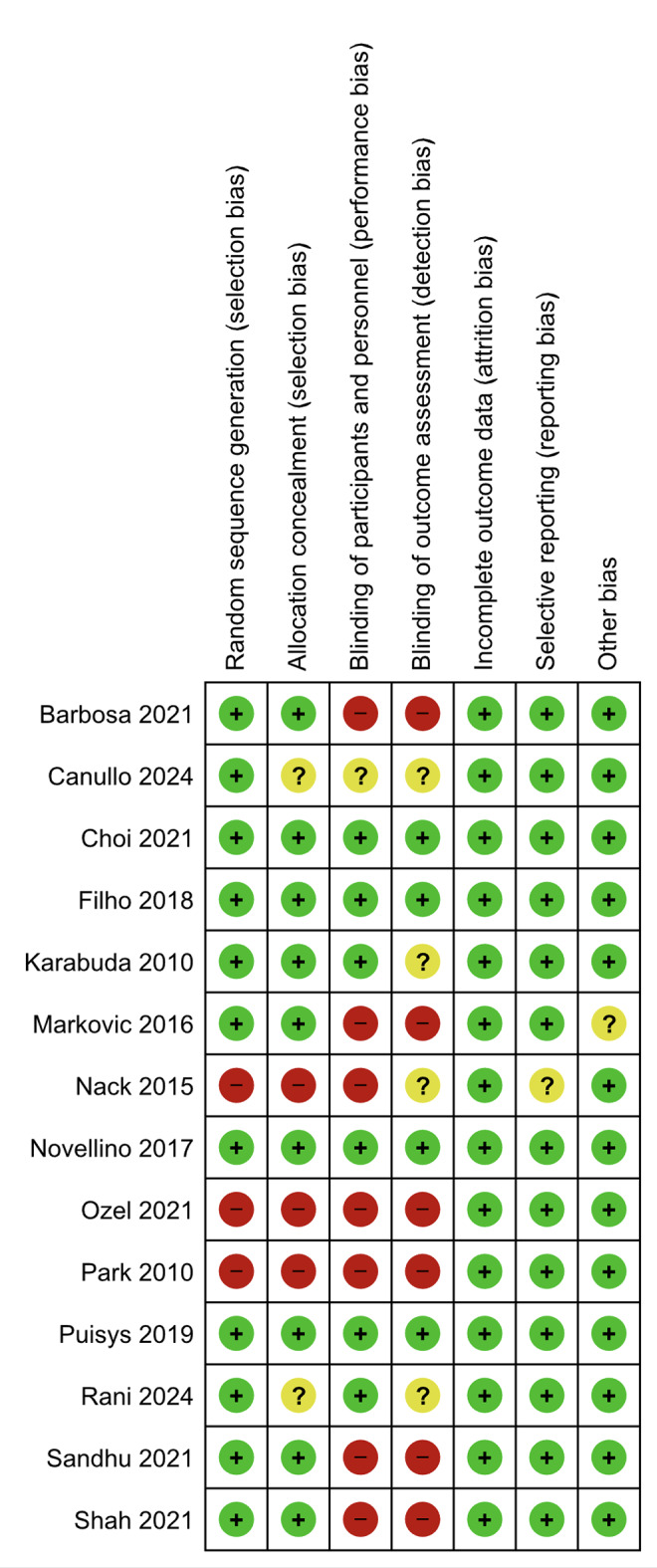
Risk of bias summary, showing the bias in each item for individual studies.

## References

[ref1] Αlmassri HNS, Ma Y, Dan Z, Ting Z, Cheng Y, Wu X (2020). Implant stability and survival rates of a hydrophilic versus a conventional sandblasted, acid-etched implant surface: Systematic review and meta-analysis. J Am Dent Assoc.

[ref2] Barbosa PP, Cruvinel TM, Sakakura CE, Pimentel Lopes de Oliveira GJ, Zuza EC (2021). Primary and secondary stability of implants with hydrophilic surfaces in the posterior maxilla: a split-mouth randomized controlled clinical trial. Int J Oral Maxillofac Implants.

[ref3] Bornstein MM, Wittneben JG, Brägger U, Buser D (2010). Early loading at 21 days of non-submerged titanium implants with a chemically modified sandblasted and acid-etched surface: 3-year results of a prospective study in the posterior mandible. J Periodontol.

[ref4] Bornstein MM, Schmid B, Belser UC, Lussi A, Buser D (2005). Early loading of non-submerged titanium implants with a sandblasted and acid-etched surface. 5-year results of a prospective study in partially edentulous patients. Clin Oral Implants Res.

[ref5] Bornstein MM, Hart CN, Halbritter SA, Morton D, Buser D (2009). Early loading of nonsubmerged titanium implants with a chemically modified sand-blasted and acid-etched surface: 6-month results of a prospective case series study in the posterior mandible focusing on peri-implant crestal bone changes and implant stability quotient (ISQ) values. Clin Implant Dent Relat Res.

[ref6] Bosshardt DD, Salvi GE, Huynh-Ba G, Ivanovski S, DonosN, Lang NP (2011). The role of bone debris in early healing adjacent to hydrophilic and hydrophobic implant surfaces in man. Clin Oral Implants Res.

[ref9] Canullo L, Menini M, Covani U, Pesce P (2020). Clinical outcomes of using a prosthetic protocol to rehabilitate tissue-level implants with a convergent collar in the esthetic zone: A 3-year prospective study. J Prosthet Dent.

[ref10] Canullo L, Menini M, Pesce P, Iacono R, Sculean A, Del Fabbro M (2024). Nano-superhydrophilic and bioactive surface in poor bone environment. Part 1: transition from primary to secondary stability. A controlled clinical trial : Bioactive implant surfaces in poor density bone. Clin Oral Investig 2024 14;28(7):372. Erratum in: Clin Oral Investig.

[ref11] Canullo L, Pesce P, Patini R, Antonacci D, Tommasato G (2020). What are the effects of different abutment morphologies on peri-implant hard and soft tissue behavior? A systematic review and meta-analysis. Int J Prosthodont.

[ref12] Cassetta M, Brandetti G, Altieri F (2022). Are the insertion torque value and implant stability quotient correlated, and if so, can insertion torque values predict secondary implant stability? A prospective parallel cohort study. Int J Oral Maxillofac Implants.

[ref13] Chambrone L, Shibli JA, Mercúrio CE, Cardoso B, Preshaw PM (2015). Efficacy of standard (SLA) and modified sandblasted and acid-etched (SLActive) dental implants in promoting immediate and/or early occlusal loading protocols: a systematic review of prospective studies. Clin Oral Implants Res.

[ref14] Choi B, Lee YC, Oh KC, Lee JH (2021). Effects of photofunctionalization on early osseointegration of titanium dental implants in the maxillary posterior region: a randomized double-blinded clinical trial. Int J Implant Dent.

[ref16] Filippi A, Higginbottom FL, Lambrecht T, Levin BP, Meier JL, Rosen PS (2013). A prospective noninterventional study to document implant success and survival of the Straumann Bone Level SLActive dental implant in daily dental practice. Quintessence Int.

[ref17] Funato A, Ogawa T (2013). Photofunctionalized dental implants: a case series in compromised bone. Int J Oral Maxillofac Implants.

[ref18] Funato A, Yamada M, Ogawa T (2013). Success rate, healing time, and implant stability of photofunctionalized dental implants. Int J Oral Maxillofac Implants.

[ref19] Ganeles J, Zöllner A, Jackowski J, ten Bruggenkate C, Beagle J, Guerra F (2008). Immediate and early loading of Straumann implants with a chemically modified surface (SLActive) in the posterior mandible and maxilla: 1-year results from a prospective multicenter study. Clin Oral Implants Res.

[ref20] Gil J, Pérez R, Herrero-Climent M, Rizo-Gorrita M, Torres-Lagares D, Gutierrez JL (2021). Benefits of residual aluminum oxide for sand blasting titanium dental implants: osseointegration and bactericidal effects. Materials (Basel).

[ref22] Guler AU, Sumer M, Duran I, Sandikci EO, Telcioglu NT (2013). Resonance frequency analysis of 208 Straumann dental implants during the healing period. J Oral Implantol.

[ref23] Heimes D, Becker P, Pabst A, Smeets R, Kraus A, Hartmann A (2023). How does dental implant macrogeometry affect primary implant stability? A narrative review. Int J Implant Dent.

[ref24] Hicklin SP, Janner SF, Schnider N, Chappuis V, Buser D, Brägger U (2020). Early loading of titanium dental implants with an intraoperatively conditioned hydrophilic implant surface: 3-year results of a prospective case series study. Int J Oral Maxillofac Implants.

[ref25] Hicklin SP, Schneebeli E, Chappuis V, Janner SF, Buser D, Brägger U (2016). Early loading of titanium dental implants with an intra-operatively conditioned hydrophilic implant surface after 21 days of healing. Clin Oral Implants Res.

[ref27] Hirota M, Ozawa T, Iwai T, Ogawa T, Tohnai I (2018). Effect of photofunctionalization on early implant failure. Int J Oral Maxillofac Implants.

[ref28] Hirota M, Ozawa T, Iwai T, Ogawa T, Tohnai I (2016). Implant stability development of photofunctionalized implants placed in regular and complex cases: A case-control study. Int J Oral Maxillofac Implants.

[ref29] Hirota M, Ozawa T, Iwai T, Ogawa T (2020). UV-mediated photofunctionalization of dental implant: A seven-year results of a prospective study. J Clin Med.

[ref30] Hyzy SL, Cheng A, Cohen DJ, Yatzkaier G, Whitehead AJ, Clohessy RM (2016). Novel hydrophilic nanostructured microtexture on direct metal laser sintered Ti-6Al-4V surfaces enhances osteoblast response in vitro and osseointegration in a rabbit model. J Biomed Mater Res A.

[ref31] Illing B, Mohammadnejad L, Theurer A, Schultheiss J, Kimmerle-Mueller E, Rupp F (2023). Biological performance of titanium surfaces with different hydrophilic and nanotopographical features. Materials (Basel).

[ref32] Insua A, Galindo-Moreno P, Miron RJ, Wang HL, Monje A (2024). Emerging factors affecting peri-implant bone metabolism. Periodontol 2000.

[ref33] Ivanovski S, Lee RSB, Fernandez-Medina T, Pinto N, Andrade C, Quirynen M (2025). Impact of autologous platelet concentrates on the osseointegration of dental implants. Periodontol 2000.

[ref34] Karabuda ZC, Abdel-Haq J, Arisan V (2011). Stability, marginal bone loss and survival of standard and modified sand-blasted, acid-etched implants in bilateral edentulous spaces: a prospective 15-month evaluation. Clin Oral Implants Res.

[ref36] Kido D, Komatsu K, Suzumura T, Matsuura T, Cheng J, Kim J (2023). Influence of surface contaminants and hydrocarbon pellicle on the results of wettability measurements of titanium. Int J Mol Sci.

[ref37] Kitajima H, Ogawa T (2016). The use of photofunctionalized implants for low or extremely low primary stability cases. Int J Oral Maxillofac Implants.

[ref38] Lang NP, Salvi GE, Huynh-Ba G, Ivanovski S, Donos N, Bosshardt DD (2011). Early osseointegration to hydrophilic and hydrophobic implant surfaces in humans. Clin Oral Implants Res.

[ref39] López-Valverde N, Flores-Fraile J, Ramírez JM, Sousa BM, Herrero-Hernández S, López-Valverde A (2020). Bioactive surfaces vs. conventional surfaces in titanium dental implants: a comparative systematic review. J Clin Med.

[ref40] Luke Yeo IS (2022). Dental implants: enhancing biological response through surface modifications. Dent Clin North Am.

[ref41] Luongo G, Oteri G (2010). A noninterventional study documenting use and success of implants with a new chemically modified titanium surface in daily dental practice. J Oral Implantol.

[ref43] Marković A, Čolić S, Šćepanović M, Mišić T, Ðinić A, Bhusal DS (2015). A 1-year prospective clinical and radiographic study of early-loaded bone level implants in the posterior maxilla. Clin Implant Dent Relat Res.

[ref44] Marković A, Đinić A, Calvo Guirado JL, Tahmaseb A, Šćepanović M, Janjić B (2017). Randomized clinical study of the peri-implant healing to hydrophilic and hydrophobic implant surfaces in patients receiving anticoagulants. Clin Oral Implants Res.

[ref46] Morton D, Bornstein MM, Wittneben JG, Martin WC, Ruskin JD, Hart CN (2010). Early loading after 21 days of healing of nonsubmerged titanium implants with a chemically modified sandblasted and acid-etched surface: two-year results of a prospective two-center study. Clin Implant Dent Relat Res.

[ref47] Nack C, Raguse JD, Stricker A, Nelson K, Nahles S (2015). Rehabilitation of irradiated patients with chemically modified and conventional SLA implants: five-year follow-up. J Oral Rehabil.

[ref48] Nicolau P, Guerra F, Reis R, Krafft T, Benz K, Jackowski J (2019). 10-year outcomes with immediate and early loaded implants with a chemically modified SLA surface. Quintessence Int.

[ref49] Nicolau P, Korostoff J, Ganeles J, Jackowski J, Krafft T, Neves M (2013). Immediate and early loading of chemically modified implants in posterior jaws: 3-year results from a prospective randomized multicenter study. Clin Implant Dent Relat Res.

[ref50] Novellino MM, Sesma N, Zanardi PR, Laganá DC (2017). Resonance frequency analysis of dental implants placed at the posterior maxilla varying the surface treatment only: A randomized clinical trial. Clin Implant Dent Relat Res.

[ref51] Ogawa T (2014). Ultraviolet photofunctionalization of titanium implants. Int J Oral Maxillofac Implants.

[ref52] Papaspyridakos P, Chen CJ, Singh M, Weber HP, Gallucci GO (2012). Success criteria in implant dentistry: a systematic review. J Dent Res.

[ref53] Park JC, Ha SR, Kim SM, Kim MJ, Lee JB, Lee JH (2010). A randomized clinical 1-year trial comparing two types of non-submerged dental implants. Clin Oral Implants Res.

[ref54] Pesce P, Menini M, Santori G, Giovanni E, Bagnasco F, Canullo L (2020). Photo and plasma activation of dental implant titanium surfaces. a systematic review with meta-analysis of pre-clinical studies. J Clin Med.

[ref55] Puisys A, Schlee M, Linkevicius T, Petrakakis P, Tjaden A (2020). Photo-activated implants: a triple-blinded, split-mouth, randomized controlled clinical trial on the resistance to removal torque at various healing intervals. Clin Oral Investig.

[ref57] Rosas-Díaz JC, Malpartida-Carrillo V, Córdova-LimayllaNE, Guerrero ME, Palomino-Zorrilla JJ, Cervantes-Ganoza LA (2022). Resonance frequency analysis mapping during implant healing using a nanostructured hydroxyapatite surface. J Int Soc Prev Community Dent.

[ref58] Roy M, Pompella A, Kubacki J, Szade J, Roy RA, HedzelekW (2016). Photofunctionalization of titanium: an alternative explanation of its chemical-physical mechanism. PLoS One.

[ref59] Rupp F, Gittens RA, Scheideler L, Marmur A, Boyan BD, Schwartz Z (2014). A review on the wettability of dental implant surfaces I: theoretical and experimental aspects. Acta Biomater.

[ref60] Rupp F, Liang L, Geis-Gerstorfer J, Scheideler L, Hüttig F (2018). Surface characteristics of dental implants: A review. Dent Mater.

[ref61] Sayin Ozel G, Inan O, Secilmis Acar A, Alniacik IyidoganG, Dolanmaz D, Yildirim G (2021). Stability of dental implants with sandblasted and acid-etched (SLA) and modified (SLActive) surfaces during the osseointegration period. J Dent Res Dent Clin Dent Prospects.

[ref62] Sandhu R, Kheur M, Lakha T, Kheur S, Le B (2021). Comparative evaluation of implant stability quotient trends, crestal bone loss and survival of photofunctionalised and untreated dental implants: A split-mouth randomised controlled clinical trial. Int J Oral Implantol.

[ref63] Şener-Yamaner ID, Yamaner G, Sertgöz A, Çanakçi CF, Özcan M (2017). Marginal bone loss around early-loaded sla and slactive implants: radiological follow-up evaluation up to 6.5 years. Implant Dent.

[ref64] Schupbach P, Glauser R, Bauer S (2019). Al2O3 particles on titanium dental implant systems following sandblasting and acid-etching process. Int J Biomater.

[ref65] Schwarz F, Wieland M, Schwartz Z, Zhao G, Rupp F, Geis-Gerstorfer J (2009). Potential of chemically modified hydrophilic surface characteristics to support tissue integration of titanium dental implants. J Biomed Mater Res B Appl Biomater.

[ref66] Shah SA, Singh BP, Rao J, Kumar L, Singh M, Singh PK (2021). Biological and esthetic outcome of immediate dental implant with the adjunct pretreatment of immediate implants with platelet-rich plasma or photofunctionalization: A randomized controlled trial. J Indian Prosthodont Soc.

[ref67] Sivaswamy V, Bahl V (2023). Surface modifications of commercial dental implant systems: an overview. J Long Term Eff Med Implants.

[ref68] Stricker A, Voss PJ, Gutwald R, Schramm A, Schmelzeisen R (2003). Maxillary sinus floor augmention with autogenous bone grafts to enable placement of SLA-surfaced implants: preliminary results after 15–40 months. Clin Oral Implants Res.

[ref69] Suzumura T, Matsuura T, Komatsu K, Ogawa T (2023). A novel high-energy vacuum ultraviolet light photofunctionalization approach for decomposing organic molecules around titanium. Int J Mol Sci.

[ref70] Tomar S, Saxena D, Kaur N (2025). Marginal bone loss around implants with platform switching and platform matched connection: A systematic review. J Prosthet Dent.

[ref71] Tomasi C, Derks J (2022). Etiology, occurrence, and consequences of implant loss. Periodontol 2000.

[ref72] Wallkamm B, Ciocco M, Ettlin D, Syfrig B, Abbott W, Listrom R, Levin BP, Rosen PS (2015). Three-year outcomes of Straumann Bone Level SLActive dental implants in daily dental practice: a prospective non-interventional study. Quintessence Int.

[ref73] Zhang C, Zhang T, Geng T, Wang X, Lin K, Wang P (2021). Dental implants loaded with bioactive agents promote osseointegration in osteoporosis: a review. Front Bioeng Biotechnol.

[ref74] Zöllner A, Ganeles J, Korostoff J, Guerra F, Krafft T, Brägger U (2008). Immediate and early non-occlusal loading of Straumann implants with a chemically modified surface (SLActive) in the posterior mandible and maxilla: interim results from a prospective multicenter randomized-controlled study. Clin Oral Implants Res.

